# Implementation research: a mentoring programme to improve laboratory quality in Cambodia

**DOI:** 10.2471/BLT.15.163824

**Published:** 2016-08-30

**Authors:** Lucy A Perrone, Vireak Voeurng, Sophat Sek, Sophanna Song, Nora Vong, Chansamrach Tous, Jean-Frederic Flandin, Deborah Confer, Alexandre Costa, Robert Martin

**Affiliations:** a International Training and Education Center for Health, Department of Global Health, School of Public Health, University of Washington, 901 Boren Ave, Suite 1100, Seattle, WA 98104, United States of America.; b International Training and Education Center for Health-Cambodia, Phnom Penh, Cambodia.; c World Health Organization, Phnom Penh, Cambodia.

## Abstract

**Objective:**

To implement a mentored laboratory quality stepwise implementation (LQSI) programme to strengthen the quality and capacity of Cambodian hospital laboratories.

**Methods:**

We recruited four laboratory technicians to be mentors and trained them in mentoring skills, laboratory quality management practices and international standard organization (ISO) 15189 requirements for medical laboratories. Separately, we trained staff from 12 referral hospital laboratories in laboratory quality management systems followed by tri-weekly in-person mentoring on quality management systems implementation using the LQSI tool, which is aligned with the ISO 15189 standard. The tool was adapted from a web-based resource into a software-based spreadsheet checklist, which includes a detailed action plan and can be used to qualitatively monitor each laboratory’s progress. The tool – translated into Khmer – included a set of quality improvement activities grouped into four phases for implementation with increasing complexity. Project staff reviewed the laboratories’ progress and challenges in weekly conference calls and bi-monthly meetings with focal points of the health ministry, participating laboratories and local partners. We present the achievements in implementation from September 2014 to March 2016.

**Findings:**

As of March 2016, the 12 laboratories have completed 74–90% of the 104 activities in phase 1, 53–78% of the 178 activities in phase 2, and 18–26% of the 129 activities in phase 3.

**Conclusion:**

Regular on-site mentoring of laboratories using a detailed action plan in the local language allows staff to learn concepts of quality management system and learn on the job without disruption to laboratory service provision.

## Résumé

**Objectif:**

Mettre en place un programme encadré de mise en œuvre par étape du système de gestion de la qualité au laboratoire (LQSI) afin d'améliorer la qualité et les capacités des laboratoires hospitaliers du Cambodge.

**Méthodes:**

Nous avons recruté quatre techniciens de laboratoire devant assurer le rôle de mentors et les avons formés au mentorat, aux pratiques de gestion de la qualité au laboratoire ainsi qu'aux exigences de la norme ISO 15189 concernant les laboratoires d'analyses médicales. D'autre part, nous avons formé du personnel de 12 laboratoires d'hôpitaux de premier niveau aux systèmes de gestion de la qualité au laboratoire, suivis par des activités trihebdomadaires de mentorat en face à face sur la mise en œuvre de systèmes de gestion de la qualité à l'aide de l'outil LQSI, dans le respect de la norme ISO 15189. Cet outil, à l'origine une ressource en ligne, a été adapté sous forme de tableur en une liste de contrôle qui comprend un plan d'action détaillé et peut être utilisée pour contrôler, du point de vue qualitatif, la progression de chaque laboratoire. Traduit en khmer, il comprenait une série d'activités visant à améliorer la qualité, regroupées en quatre phases de mise en œuvre à complexité croissante. L'équipe du projet a examiné la progression et les difficultés des laboratoires lors de conférences téléphoniques hebdomadaires et de réunions bimensuelles avec les référents du ministère de la Santé, des laboratoires participants et des partenaires locaux. Nous présentons les résultats du programme de septembre 2014 à mars 2016.

**Résultats:**

Au mois de mars 2016, les 12 laboratoires ont effectué 74 à 90% des 104 activités de la phase 1, 53 à 78% des 178 activités de la phase 2, et 18 à 26% des 129 activités de la phase 3.

**Conclusion:**

Un mentorat régulier sur place à l'aide d'un plan d'action détaillé dans la langue locale permet au personnel des laboratoires d'acquérir les notions de système de gestion de la qualité et d'apprendre tout en travaillant, et ceci sans interruption des services des laboratoires.

## Resumen

**Objetivo:**

Implementar un programa orientado de implementación gradual de la calidad de los laboratorios (LQSI, por sus siglas en inglés) para mejorar la calidad y la capacidad de los laboratorios de los hospitales de Camboya.

**Métodos:**

Se contrataron cuatro técnicos de laboratorio para que fuesen mentores y se les formó en habilidades de orientación, prácticas de gestión de la calidad de los laboratorios y los requisitos de la organización internacional de la normalización (ISO) 15189 para laboratorios médicos. Por otro lado, se formó a personal de 12 laboratorios hospitalarios de referencia en sistemas de gestión de la calidad de los laboratorios, además de proporcionarles orientación presencial cada tres semanas acerca de la implementación de sistemas de gestión de la calidad mediante la herramienta LQSI, que sigue la norma ISO 15189. La herramienta se ha adaptado de un recurso basado en Internet a una lista de verificación de hoja de cálculo basada en un programa, la cual incluye un plan de acción detallado y puede utilizarse para controlar de forma cualitativa el progreso del laboratorio. La herramienta, traducida al jemer, incluye un conjunto de actividades de mejora de la calidad agrupadas en cuatro fases, las cuales se implementarán con cada vez una mayor complicación. El personal del proyecto analizó el progreso de los laboratorios, así como los desafíos, en videoconferencias semanales y reuniones bimensuales con puntos centrales del ministerio de sanidad, los laboratorios participantes y socios locales. Se presentan los logros en la implementación de septiembre de 2014 a marzo de 2016.

**Resultados:**

En marzo de 2016, los 12 laboratorios han completado entre el 74 y el 90% de las 104 actividades de la fase 1, entre el 53 y el 78% de las 178 actividades de la fase 2 y entre el 18 y el 26% de las 129 actividades de la fase 3.

**Conclusión:**

La orientación regular de los laboratorios in situ mediante la utilización de un plan de acción detallado en el idioma local permite que el personal aprenda conceptos de sistema de gestión de la calidad y aprenda sus funciones sin interrumpir el servicio del laboratorio.

## ملخص

**الغرض:**

تطبيق برنامج توجيهي لتنفيذ إجراءات تحسين جودة المختبرات تدريجيًا (LQSI) بغرض رفع جودة المختبرات في المستشفيات الكمبودية وزيادة قدرة استيعابها للحالات.

**الطريقة:**

لقد قمنا بانتداب أربعة من فنيي المختبرات ليكونوا موجهين وقمنا بتدريبهم على المهارات التوجيهية، وممارسات إدارة جودة المختبرات، ومتطلبات المعيار 15189 من المنظمة الدولية للمعايير (ISO) للمختبرات الطبية. وعلى جانب منفصل، قمنا بتدريب طاقم يضم أعضاءً من 12 مختبرًا في المستشفيات المحالين إليها على استخدام أنظمة إدارة جودة المختبرات وأعقبه تطبيق برنامج توجيهي شخصي ثلاث مرات أسبوعيًا بخصوص تنفيذ أنظمة إدارة الجودة باستخدام أداة LQSI "تنفيذ إجراءات تحسين جودة المختبرات تدريجيًا"، والتي تتوافق مع المعيار 15189 الخاص بالمنظمة الدولية للمعايير. لقد تمت تهيئة الأداة من أحد موارد الإنترنت وتحويلها إلى قائمة فحص جدولية برمجية، والتي تشمل خطة عمل تفصيلية ويمكن استخدامها لمراقبة التقدم الخاص بكل مختبر نوعيًا. تضمنت الأداة التي تمت ترجمتها إلى اللغة الخميرية (اللغة الكمبودية) مجموعة من أنشطة تحسين الجودة والتي تم تجميعها في إطار أربع مراحل تنفيذية مع زيادة مستوى تعقيدها. قام طاقم العاملين في المشروع بمراجعة مستوى التقدم بالمختبرات وطبيعة التحديات التي تواجهها من خلال مجموعة من الاتصالات الجماعية الأسبوعية واجتماعين شهريين مع مراكز التنسيق في وزارة الصحة، والمختبرات المشاركة، والشركاء المحليين. نحن نقدم الإنجازات التي تحققت في عمليات التنفيذ في الفترة من شهر سبتمبر/أيلول من عام 2014 وحتى شهر مارس/آذار من عام 2016.

**النتائج:**

ابتداءً من شهر مارس/آذار من عام 2016، قام العاملون في المختبرات الـ 12 باستكمال نسبة تتراوح من 74 إلى 90‏% من 104 أنشطة في المرحلة الأولى، ونسبة تتراوح من 53 إلى 78‏% من 178 نشاطًا في المرحلة الثانية، ونسبة تتراوح من 18 إلى ‏26‏% من 129 نشاطًا في المرحلة الثالثة.

**الاستنتاج:**

إن المراقبة المنتظمة على المختبرات في موقع العمل باستخدام خطة عمل تفصيلية باللغة المحلية يتيح للطاقم تعلّم مفاهيم نظام إدارة الجودة والتعلّم من خلال التدريب العملي دون الإخلال بمهام توفير الخدمات المختبرية.

## 摘要

**目的:**

旨在实施一项指导性实验室质量逐步实施 (LQSI) 计划，以提高柬埔寨医院实验室的质量和能力。

**方法:**

我们招募了四位实验室技术人员作为指导员，并且在指导技能、实验室质量管理实务和国际标准组织 (ISO) 15189 医学实验室要求方面对他们进行了培训。 另外，我们还在实验室质量管理体系中培训了 12 位转诊医院实验室的工作人员，随后使用符合 ISO 15189 标准的 LQSI 工具针对质量管理系统实施每三周进行一次现场指导。 该工具从基于网络的资源改编成基于软件的电子表格清单，其中包括详细的行动计划并且可用于定性监测各个实验室的进展。 该工具（已翻译成高棉语）包括一系列质量改进活动，分四个实施阶段，复杂度不断提高。 在每周一次的电话会议和每两个月一次的座谈会中，项目工作人员与卫生部归口单位、参与实验室以及当地的合作伙伴一起审查了实验室的进展和挑战。 我们介绍了从 2014 年 9 月至 2016 年 3 月的实施成果。

**结果:**

截至 2016 年 3 月，12 个实验室在第 1 阶段完成了 104 项活动的 74–90%；在第 2 阶段完成了 178 项活动的 53–78%；在第 3 阶段完成了 129 项活动的 18–26%。

**结论:**

通过使用详细的行动计划以当地语言进行定期实验室现场指导，让工作人员学习质量管理体系的概念，并且在工作中进行学习，从而无需中断实验室提供的服务。

## Резюме

**Цель:**

Реализовать программу поэтапного улучшения качества работы лабораторий (LQSI) при поддержке наставников для повышения качества работы и производительности медицинских лабораторий в Камбодже.

**Методы:**

Для выполнения функции наставников были привлечены четыре техника-лаборанта, которых обучили навыкам наставничества, управлению качеством работы лаборатории и требованиям для медицинских лабораторий Международной организации по стандартизации (ИСО) 15189. Кроме того, была проведена подготовка персонала 12 лабораторий лечебно-диагностических центров по теме «Системы управления качеством работы лаборатории», после чего каждую третью неделю при непосредственном участии наставников происходило внедрение систем управления качеством с помощью инструмента LQSI, приведенного в соответствие со стандартом ИСО 15189. Этот инструмент был адаптирован на основе веб-ресурса в программную динамическую таблицу, включающую подробный план действий, который был использован для отслеживания качественных показателей развития каждой лаборатории. Инструмент был переведен на кхмерский язык и включал комплекс мероприятий по улучшению качества, сгруппированных в четыре фазы с последовательным увеличением сложности внедрения. Персонал проекта анализировал развитие лабораторий и проблемы, с которыми они сталкивались, на еженедельном селекторном совещании и проводимом каждые два месяца собрании при активном внимании со стороны министерства здравоохранения, задействованных лабораторий и местных партнеров. В статье приводятся достижения в области внедрения за период с сентября 2014 года по март 2016 года.

**Результаты:**

По состоянию на март 2016 года все 12 лабораторий завершили 74–90% из 104 мероприятий в фазе 1, 53–78% из 178 мероприятий в фазе 2 и 18–26% из 129 мероприятий в фазе 3.

**Вывод:**

Регулярное обучение персонала лабораторий методом очного наставничества при использовании подробного плана действий на местном языке позволяет научить сотрудников принципам системы управления качеством на рабочем месте без отрыва от оказания лабораторных услуг.

## Introduction

The development of functional laboratory systems is a critical component of sustainable health systems,^1^^,^^2^ and a key requirement for countries to meet international health regulations requirements and strategic global health goals.^3^^,^^4^ The rapid emergence of infectious diseases – such as Ebola virus disease – highlights the need for stronger health systems including capable and sustainable laboratories in nations where the risk of zoonotic and epidemic-prone infectious diseases remains a threat.^5^^,^^6^ The prevention, detection and response to disease outbreaks of international concern require that laboratories produce accurate and reliable test results. However, many laboratories in countries with constrained resources lack the capacity to detect pathogens of national and international concern and provide poor quality supportive testing that is often unreliable and untimely.^4^

Inaccuracies in diagnostic testing can lead to potentially devastating outcomes for patient and public health, compromise the quality of surveillance data and can ultimately affect health policy.^7^^–^^10^ Most laboratory diagnostic errors happen in the pre-analytic phase (32–75%), while 13–32% occur in the analytic phase and 9–31% in the post-analytic phase.^11^^,^^12^ The frequency of diagnostic errors can be as high as one for every 330 tests^13^ with 25% of such errors producing a major impact on patient care due to test repetition, inappropriate investigations or even unjustified clinical and therapeutic management.^14^ A review of data from external quality assurance and proficiency testing schemes conducted in 30 national reference and provincial referral level hospital laboratories in Cambodia in 2014 showed that while testing in microbiology and serology units have met acceptable levels (accuracy scores > 90%), haematology and clinical chemistry results remain extremely poor (< 50% accuracy).^15^^,^^16^ Hence there is a need to address the quality of testing for haematology and clinical chemistry to ensure appropriate patient care.^15^^,^^16^

Between 2013 and 2014, the Cambodian Ministry of Health, World Health Organization (WHO), United States Centers for Disease Control and Prevention (CDC) and the Integrated Quality Laboratory Services company conducted a capacity assessment of 28 public hospital laboratories across Cambodia. The evaluators used a modified WHO laboratory facility assessment tool which assesses laboratory capacity in 11 areas of operations and international health regulation preparedness.^17^ Average general indicator scores (a measure of laboratory capacity) for these laboratories ranged from 36 to 60%. The result also showed that most public hospital laboratories do not have a quality management system in place to ensure the quality of diagnostic testing nor the capacity to meet international health regulation requirements and population health demands. Specific challenges that needed to be addressed included the lack of management oversight, lack of training and awareness of quality control procedures, unstable power supply, poor quality reagents and supplies, lack of standard management guidelines for supplies and equipment, and lack of equipment standardization between laboratories and local technical capacity for equipment, calibration, repair and maintenance. In addition, there have been limited public financial resources allocated to purchase and maintain laboratory equipment and reagents.

Laboratory diagnostics is a multifaceted activity, involving a complex variety of technologies, processes and personnel. An effective way to strengthen clinical laboratory practice is to implement a quality management system that is aligned with international quality standards,^10^^,^^18^^,^^19^ which focuses on key operational areas in the laboratory: organization, personnel, equipment, purchasing and inventory, process, information management, documents and records, occurrence management, assessment, process improvement, customer service, and facilities and safety. In 2011, CDC began assisting the Cambodian health ministry by implementing the strengthening laboratory management towards accreditation (SLMTA) programme in 12 hospital laboratories in the country.^20^ The programme used short courses and work-based improvement projects supported by site visits by mentors to teach quality management system principles to laboratory staff. While some participating laboratories demonstrated improvements in testing accuracy, timeliness and reliability, there remained a need to scale up laboratory quality improvement efforts across Cambodia and to dedicate more resources to training and staff mentoring in quality management.^21^^–^^24^ The mentored laboratory quality stepwise implementation (LQSI) programme described in this paper began in 2014 and is being implemented in additional 12 national and provincial referral hospital laboratories in Cambodia. The programme aimed to expand national coverage of quality management system training and implementation.

## Methods

### Setting

This non-randomized, quasi-experimental quantitative study was done in Cambodia, which has over 15 million people living in 25 provinces. The national health system has a tiered structure, ordered from national to peripheral levels, which addresses curative and preventive health services. The 18 public tertiary level referral hospitals serve as a central hub for health care; however, testing services and capacity at many of these laboratories are limited to less than 10 tests (Table 1).

**Table 1:** Capacities of the hospitals and their laboratories chosen from the mentored laboratory quality stepwise implementation programme, Cambodia, 2014–2016

Cohort no.	Hospital	Mean no. of OPD visits/day	No. of beds (mean % occupancy/month)	Laboratory sections											No. of tests/week^f^	2013 overall laboratory capacity score in %^g^
				Hematology^a^	Biochemistry^b^	Serology^c^	Microbiology^d^	Parasitiology^e^	Blood bank	Tuberculosis				Urine		
										SM	RD	MGIT	DST			
1	National Paediatric	230	150 (58)	Yes	Yes	No	Yes	Yes	No	Yes	No	No	No	Yes	1650	54
	Preah Kossamak	130	250 (63)	Yes	Yes	Yes	Yes	Yes	No	Yes	No	No	No	Yes	761	46
	Kampong Cham	180	260 (91)	Yes	Yes	Yes	Yes	No	No	Yes	Yes	Yes	Yes	No	800	58
	Svay Rieng	47	168 (83)	Yes	Yes	Yes	Yes	Yes	No	Yes	Yes	No	No	Yes	1140	69
	Takeo	41	250 (100)	Yes	Yes	Yes	Yes	Yes	No	Yes	No	No	No	Yes	1815	57
	Kampot	9	67 (43)	Yes	Yes	Yes	Yes	No	No	Yes	Yes	No	No	No	338	57
2	Ang Duong	16	98 (79)	Yes	Yes	Yes	No	Yes	No	No	No	No	No	Yes	700	33
	Khmer Soviet Friendship	678	500 (115)^h^	Yes	Yes	Yes	Yes	No	Yes	No	No	No	No	No	7881	60
	Sihanoukville	22	120 (85)	Yes	Yes	Yes	No	Yes	Yes	Yes	No	No	No	Yes	495	43
	Kratie	20	150 (65)	Yes	Yes	Yes	Yes	No	Yes	No	Yes	No	No	Yes	148	48
	Prey Veng	90	106 (102)^h^	Yes	Yes	Yes	Yes	No	Yes	Yes	Yes	No	No	Yes	741	44
	Kandal	22	190 (65)	Yes	Yes	Yes	No	Yes	No	Yes	Yes	No	No	Yes	740	40

DST: drug susceptibility testing; MGIT: Mycobacteria growth indicator tube; OPD: outpatient department; RD: rapid diagnosis; SM: smear microscopy.

^a^ Includes **complete blood count** and blood smear.

^b^ Includes analyses of uric acid, albumin, amylase, bilirubin, calcium, cholesterol, creatinine, etc.

^c^ Includes tests for human immunodeficiency virus 1/2 antibody (ab), hepatitis (hep) B antigen (ag), hep B ab, hep C ab, typhoid, pregnancy test, syphilis ab. which were mainly rapid tests. Khmer Soviet Friendship Hospital laboratory can perform the HBV5 test for hep B, which detects HBsAg, HBsAb, HBeAg, HBeAb, and HBcAb in one test.

^d^ Includes bacterial culture.

^e^ Includes fecal smear and blood film.

^g^ Average number of tests per week (calculated from September 2014 to June 2015, cumulative for all laboratory sections).

^g^ Average laboratory capacity scores were based on evaluations of 11 areas of laboratory operations using the World Health Organization laboratory quality management system toolkit.^25^

^h^ There are often more inpatients than beds available.

### Site selection

Together with the bureau of medical laboratory services, the health ministry, WHO and CDC, we selected four national and eight provincial tertiary level referral laboratories with varying patient volumes and diagnostic testing capacities for the mentored LQSI programme (Table 1). The basis of the selection was the laboratories’ service provision to key population centres, their 2013 international health regulation assessment general indicator scores and past performance in external quality assurance and proficiency testing schemes. None of the staff in the selected laboratories had quality management training before.

### Quality improvement tools

**Fig. 1:**
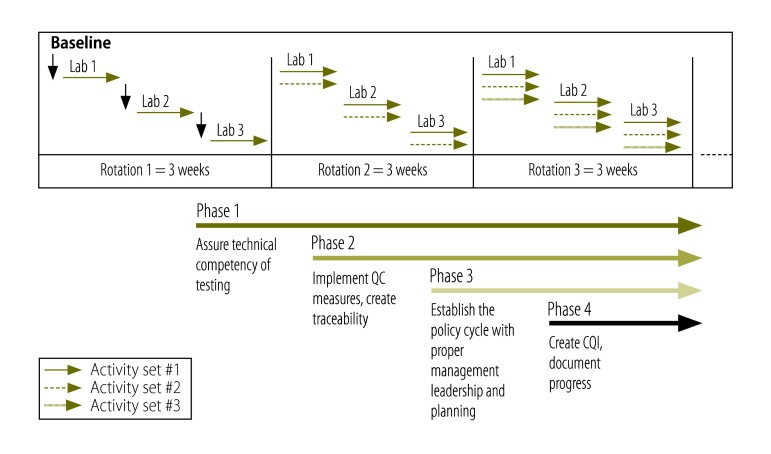
Phases of the mentored laboratory quality stepwise implementation process, Cambodia, 2014–2016

CQI: continuous quality improvement; QC: quality control.

Notes: The 465 LQSI activities are divided into four phases (phase 1 = 104 activities, phase 2 = 178 activities, phase 3 = 129 activities) and phase 4 = 54 activities). Each phase is divided further into smaller subsets of activities that laboratories address in 3-week increments. QI mentors assist laboratories to implement this subset of activities during a single rotation. QI mentors spend one week in each of their three laboratories working on these activities and any remaining activities from previous weeks.

While there are resources available for laboratory managers to assist them in implementing a quality monitoring system, many of these tools have been proprietary and thus difficult to access in resource-constrained settings. The Royal Tropical Institute in Amsterdam, the Netherlands, and WHO developed the LQSI plan and published it as an open-source web-based tool.^26^ The plan provides a stepwise guide for health laboratories to implement a quality management system that fulfils and translates the requirements of the international standard organization (ISO) 15189 standard^27^ into 465 step-by-step activities, divided in four phases, where activities for each phase are organized with increasing levels of complexity. The activities in the four phases relate to assurance of technical competency of testing (phase 1, 104 activities), implementation of quality control measures (phase 2, 178 activities), establishing a policy cycle with management, leadership and planning (phase 3, 129 activities) and creating a quality control improvement document (phase 4, 54 activities; Fig. 1).

Since most laboratories in Cambodia lack reliable Internet, we adapted the web-based tool to an Excel-based checklist (Microsoft, Redmond, United States of America), translated it into the Khmer language and further subdivided the checklist into smaller subsets of up to 30 activities that laboratories can undertake in three weeks’ time. The checklist includes an explanation of the activities, how they are aligned with ISO 15189, how to accomplish the activities, listing of the required resources for implementation, listing of staff responsible for different activities, indicators to measure completeness, estimation for required person-hours for each activity and a space to document a completeness score for each activity within each phase. Completeness scores are percentages based on the number of indicators for each activity. In addition, we developed an internal quality control monitoring tool for mentors to use during on-site visits (Box 1), which assist the laboratory staff in monitoring Levy-Jennings charts and in conducting internal control performance for quantitative tests, including the tracking of total error. We translated all instructional documents and templates produced for the laboratories into Khmer.

Box 1 **Questions in the internal quality control monitoring tool, presented by process, used in the mentored laboratory quality stepwise implementation programme, Cambodia, 2014–2016**

**Sample accessioning**

Is the laboratory rejecting samples?

Have the sample rejection forms been filled out properly?

Is the sample rejection rate (%) being calculated?^a^

Has the time of specimen receipt been recorded?

**Biochemistry**

Has the daily iQC register been completed?

Has the Levy-Jennings chart been correctly filled out?^b^

If the iQC results were out of range, which violation rules are present?^c^

Has the non-conformity corrective action form been filled out?

**Automated hematology**

Has the daily iQC register been completed?

Has the Levy-Jennings chart been correctly filled out?^b^

If the iQC results were out of range, which violation rules are present?^c^

Has the non-conformity/corrective action form been filled out?

**Serology**

Has the daily iQC register been completed?

How many times did the iQC fail?

Has the non-conformity/corrective action form been filled out?

**Reporting**

Did the laboratory report any results that were out of iQC range? In which section?

iQC: internal quality control.^a^ Information on this item is taken from mentors’ notes weekly.^b^ The mentors’ notes include mean and standard deviation calculations.^C^ Westgard rules (e.g. 0.1:3S, 2:2S, R4S, Trend, Shift etc.) were performed for all analyses.Notes: The quality improvement mentors note the laboratory’s activity on the listed iQC items on a daily and weekly basis. Information is taken daily from the mentors’ notes for five days.

### Implementation

The implementation of the mentored LQSI programme involved three stages: mentor training, laboratory staff training, and mentoring on LQSI in the laboratories. In the first stage, four trained laboratory technicians were recruited as quality improvement mentors through local human resources firms and by advertising in local newspapers. These mentors were trained in communication and mentoring skills, the ISO 15189 standard and on how to use the LQSI tool for laboratory quality improvement. In the second stage, the mentors accompanied laboratory staff from each of the 12 hospitals (three to five from each laboratory) in a weeklong training, on the principles of quality management systems, LQSI, and ISO 15189 requirements, which took place in a conference facility. Training materials were adapted from the WHO laboratory quality management system toolkit,^25^ and the workshops were conducted in Khmer and English with consecutive translation. Hospital directors as well as provincial health department directors attended the opening sessions and health ministry officials convened all workshops.

The third stage involved frequent on-site mentoring to reinforce quality management principles and practices to laboratory managers and staff in all participating laboratories. Each mentor was assigned three laboratories and rotated between them, spending one week in the laboratory during each visit (Fig. 1). Mentors continued repeating this in three-week rotation cycles, averaging 17 weeks within each laboratory, over a period of one year. Mentoring involved building close relationships with hospital leadership and staff, including directors from other departments responsible for procurement of supplies and reagents for the laboratory, and working as a quality improvement team to address challenges collectively. The mentors regularly met with laboratory managers and staff to reinforce concepts of quality and the importance of testing quality for patient outcomes. At each laboratory, the mentors assisted the laboratory managers to develop a quality improvement team consisting of a laboratory manager, a quality manager and a biosafety officer. Mentors assisted laboratory managers and staff to complete activities in each phase of the LQSI checklist, as well as to provide access to resources, templates and tools, and teach quality improvement in the laboratory. In the mentor’s absence, laboratory staff worked on the LQSI activities. The mentors encouraged laboratory staff to use quantitative quality indicator data to monitor quality improvement, such as metrics on test turnaround time and sample rejection, and tracked qualitative data such as customer feedback to motivate staff and improve communication among the hospital administrative and clinical staff. The mentors also taught and monitored laboratory staff on how to run internal quality control, plotting and analysing Levy-Jennings charts, and performing corrective actions when test runs were out of range. In each laboratory, a stock officer position was assigned and implemented an inventory management system, assessing equipment needs and updating annual operational budgets to equip laboratories appropriately. Mentors helped the laboratories coordinate with the hospital purchasing department to ensure appropriate provisioning of the laboratory, assisted laboratory staff to document the receipt of consumable supplies and monitor failures in logistics that could affect reagent quality, such as loss of cold chain. Mentors assisted laboratory managers to develop annual operational plans and budgets to equip laboratories appropriately. Equipment officers were designated at each laboratory and they completed equipment registers and implemented equipment management procedures. 

Hospital and laboratory leadership was kept informed of progress and challenges. The mentors met with hospital directors at each visit. The project team also met with the facility leadership and health ministry focal points on a quarterly basis to discuss progress and challenges. The team worked closely with other laboratory partners in the country that were involved in laboratory system strengthening. To foster inter-laboratory collaboration and collective problem solving, review meetings were convened quarterly, jointly with the 12 laboratories in the SLMTA programme. The project’s LQSI team, which included local mentors, a local project coordinator and advisors based in the United States, communicated daily and shared information and resources to implement effectively the quality management system in all 12 laboratories in an organized and systematic manner. The project coordinator reviewed the mentors progress reports daily, provided corrective action support and reviewed key documents for accuracy.

## Results

**Fig. 2:**
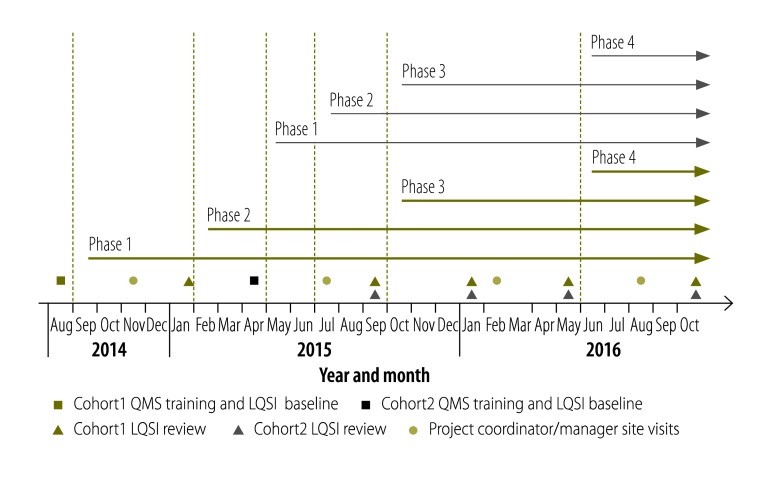
Implementation timeline of the mentored laboratory quality stepwise implementation process in Cambodia 2014–2016

LSQI: laboratory quality stepwise implementation; QMS: quality management system.

Notes: Each cohort contains six laboratories and each cohort began the LQSI process at different time. The phases represent quality improvement activities for implementation with increasing complexity. The activities in the four phases relate to assurance of technical competency of testing (phase 1), implementation of quality control measures (phase 2), establishing a policy cycle with management, leadership and planning (phase 3) and creating a quality control improvement document (phase 4).

The implementation timeline is presented in Fig. 2. Six laboratories (referred to as cohort 1) started phase 1 of the mentored LQSI programme in August 2014 and began with phase 1 baseline completeness scores ranging from 8 to 32%. As of March 2016 those six laboratories have completed 84–90% of phase 1, 68–78% of phase 2 and 22–26% of phase 3. An additional six laboratories (referred to as cohort 2) began the programme in April 2015, with phase 1 baseline scores ranging from 7 to 16% and have completed 74–85% of phase 1, 53–65% of phase 2, and 18–25% of phase 3 activities. All 12 laboratories have improved their operations in the areas of facilities and safety, organization, personnel, equipment maintenance, purchasing and inventory, testing process management, documentation and communication (Box 2). In the first 10 months of the programme, laboratories established the foundational practices of a quality management system, including establishing a documentation system to track quality indicators such as specimen rejection rate, turnaround time and client satisfaction. The programme has also improved the visibility of the laboratory within the hospitals. Clinicians and support staff have become more aware of the quality implementation efforts. Regular meetings and exchanges with leadership and management teams improved the communication between the laboratory and clinical staff. As a result, questions over test results and challenges in laboratory service provision were addressed in a timelier fashion. In addition to assisting laboratories to implement a quality management system, the mentors have helped establish quality assurance protocols and used new quality improvement tools, such as the monitoring of internal quality control procedures (Box 1) and tracking universal laboratory quality indicators.

Box 2 **Major achievements of the mentored laboratory quality stepwise implementation programme, Cambodia, 2016**

**Organization and personnel**

Each laboratory created a QMT headed by the laboratory manager, and appointed key positions of quality assurance, biosafety, equipment and stock officers. Job descriptions for all staff were developed and/or updated. Personnel folders were assembled. Organizational charts for each laboratory were updated. Authorization matrices and duty rosters were developed and implemented. Weekly meetings of the QMT were held to address LQSI and staff awareness was documented through meeting minutes. The LQSI action plan was reviewed and implemented weekly. A communication plan between the laboratory and clinical staff was used to review quality implementation progress and to discuss challenges.

**Facilities and safety**

Biosafety officers were appointed. Biosafety manuals were developed and biosafety practices were initiated at health facilities and at the process level, bio hazardous waste management and laboratory cleanliness was improved at all laboratories. Several SOPs were developed and implemented including for incident reporting. Laboratories initiated an employee health programme including vaccinations for staff and installed first aid kits in laboratories. Laboratories initiated biosecurity measures including facility access controls through structural improvements and personnel access restrictions. Laboratory managers initiated improvements to laboratory work flow by separating office space and sample collection areas from the general laboratory area.

**Equipment**

Equipment officers were appointed, laboratories completed equipment registers, established policies and SOPs for maintenance and cleaning of critical equipment. Essential equipment was placed on UPS and generator support and equipment operational needs documented. Laboratory airflow is currently being monitored. A hazardous waste material register has been completed and MSDS sheets maintained.

**Purchasing and inventory**

Stock officers appointed, developed stock inventory control register and SOPs for appropriate stock management. Laboratories have initiated procedures to verify reagent quality on newly delivered products before they are taken into service.

**Documents and records**

Quality assurance officers were appointed. Laboratories developed a master SOP and other analytical, equipment and process SOPs and initiated a document review and maintenance system. The SOPs are stored in the laboratories.

**Information management**

Laboratories improved data management processes and developed a standard format for test result reporting. SOPs were developed to ensure correct entry and verification of results on reports and improved notification processes for clinicians.

**Process control**

Laboratories initiated internal quality control procedures for each test performed, including generating Levy-Jennings charts for quantitative tests. Corrective action SOPs were used to initiate specimen acceptance or rejection processes.

**Process management**

Laboratories developed standardized laboratory test request forms, sample acceptance or rejection criteria and SOPs for reception and processing. Internal quality control registers including tracking on Levy-Jennings charts for quantitative tests were managed. Nonconformity forms were also managed and subsequent correction actions were initiated.

**Customer focus**

Laboratories developed laboratory service manuals and conducted stakeholder meetings to review population reference and critical values.

LQSI: laboratory quality stepwise implementation; MSDS: material safety data sheet; QMT: quality management team; SOP: standard operating procedure; UPS: uninterruptible power supply.Notes: Achievements as of 1 March 2016, categorized by international quality standards.^19^

## Discussion

The implementation of a quality management system in hospital laboratories is an effective method to improve laboratory-testing quality and ultimately patient care.^9^ Since 2010, 47 countries, including Cambodia, have implemented the SLMTA programme and many have reported their successes in improving laboratory quality.^28^^,^^29^In Cambodia, the need to scale up laboratory quality improvements led us to adapt the open-source LQSI tool into a quantitative checklist and combine this tool with intensive mentoring. In November 2015, a follow-up assessment in 15 laboratories across Cambodia, which also participated in a baseline assessment in 2013, was conducted by an independent consultant, who used the WHO laboratory facility assessment tool.^25^ Among these 15 laboratories, eight were sites implementing the LQSI programme (five from cohort 1 and three from cohort 2), three were implementing the SLMTA programme and four sites were controls, as they had not been implementing a quality management system programme. Laboratories implementing the LQSI programme improved their general indicator scores by 17 points compared to their baseline assessment of 2013 (mean of 69% versus 52%); among the most improved areas were quality management (+36%), data management (+29%) and specimen collection and handling (+25%). Laboratories not having received any training or support on quality management system since the baseline assessment in 2013 increased their general indicator score by only 1 percentage point (mean of 50% versus 49%). Laboratories in the SLMTA programme improved their general indicator scores by an average of 17 points (WHO Cambodia, unpublished data, January 2016). The new LQSI approach provides the global laboratory community with another method to advance laboratory quality.

Sustainable laboratory capacity strengthening is a long-term commitment that requires leadership, careful planning, effective policies and regulations and dedicated resources. In the past 25 years, many international donors have committed such resources to improve laboratory capacity, but have done so with a focus on disease specific emergencies, such as human immunodeficiency virus epidemic. However, as the 2013–2016 Ebola virus disease epidemic and other outbreaks of emerging infectious diseases have highlighted, there remains a need to improve laboratory preparedness and practice on a global scale with a focus on laboratory capacity in a non-disease specific manner.^30^ If the international health community is committed to disease detection, surveillance and pandemic preparedness, a more proactive approach is required. To meet this goal of sustainability, mentored human resource capacity building programmes will need to be implemented to train laboratory managers and staff on processes for quality laboratory services.^21^^–^^24^ Furthermore, hospital accreditation will ensure active involvement of managers and drive the need for laboratory quality, by galvanizing collective organizational commitment for quality improvement and a focus on patient-oriented thinking from managers.^28^ Resources and costs for the LQSI approach vary depending on local workforce and economy and other factors such as local government resource contribution. Typical costs include full time mentor and expatriate advisor salaries, per diem and travel support for mentor site visits, financial support for quality management system workshops and LQSI review meetings for stakeholders, quarterly reviews and mentor training and office and training supplies such as laptops, cameras, and portable digital projectors.

While the LQSI programme has been conducted with a small sample size, our intensive mentoring approach in Cambodia using the modified LQSI plan has led to faster rates of quality management system implementation than other quality management system implementation methods. We think this can be attributed to our intensive mentoring approach and the utility of the LQSI checklist, which gives partial credit for completeness towards meeting ISO 15189 requirements. This new checklist also contains a detailed action plan for laboratory managers and staff to follow to implement and maintain the quality management system. We think the LQSI action plan in the spreadsheet format is a useful tool for all health laboratories seeking ISO 15189 accreditation and we plan to make it available online free of charge. Our LQSI approach using a full-time staff of embedded quality improvement mentors has proven highly effective in implementing a quality management system in a large number of hospital laboratories in a relatively short period. Others have demonstrated that mentorship assists laboratories to implement quality improvement activities.^23^^,^^24^^,^^31^ However, we have shown how the regular presence of fully dedicated mentors and a detailed quality management system action plan in the local language improves the rate of quality management system implementation. Our successes to date can also be attributed to strong team coordination, rapid communication and collaborations including frequent in-country meetings to address challenges collectively. The LQSI review meetings also brought together key staff and leadership and provided opportunities for participants to share experiences and discuss challenges to the laboratory system.

Overall, stronger enforcement of national policies and the establishment of a legal authority over laboratory practice are needed in Cambodia. While achievements have been made, several management challenges still exist around enforcing habits of quality assurance such as rejecting inappropriate or poor quality specimens, regularly performing internal quality control, documenting tests and processes, performing corrective action, tracking quality indicators and maintenance of equipment. Only through strong leadership from hospital and laboratory directors will staff address these challenges. However, many laboratory managers and directors have assumed their positions through promotion due to their technical skills or seniority, and thus have not had formal laboratory management training. There remains a global need to improve health laboratory leadership and management for these investments in laboratory systems to be sustainable.^32^^,^^33^

In summary, classroom-based training followed by regular on-site mentoring using a detailed action plan in the local language allows staff greater opportunity to learn new concepts, ask questions and access resources leading to rapid achievements in quality management system. The mentored LQSI approach facilitates progress towards improving the accuracy, timeliness and reliability of test results in hospital laboratories and can synergize with other quality management system implementation programmes. While it may not be financially feasible for all health laboratories in Cambodia to seek full ISO 15189 accreditation, the LQSI process is a valuable undertaking for quality patient care.
